# Cognitive assessment in patients treated by immunotherapy: the prospective Cog-Immuno trial

**DOI:** 10.1186/s12885-022-10384-y

**Published:** 2022-12-13

**Authors:** Marie Lange, Bénédicte Clarisse, Alexandra Leconte, Kléouforo-Paul Dembélé, Justine Lequesne, Celeste Nicola, Martine Dubois, Laurence Derues, Yori Gidron, Hélène Castel, Florence Joly

**Affiliations:** 1 Clinical Research Department, Centre François Baclesse, 14000 Caen, France; 2 Normandie Univ, UNICAEN, INSERM, ANTICIPE, 14000 Caen, France; 3 Cancer & Cognition Platform, Ligue Contre le Cancer, 14000 Caen, France; 4 Normandie University, UNIROUEN, INSERM, U1245, Cancer and Brain Genomics, 76000 Rouen, France; 5 Institute for Research and Innovation in Biomedicine (IRIB), 76000 Rouen, France; 6 Dept. of Nursing, Faculty of Social Welfare and Health Sciences, University of Haifa, Haifa, Israel; 7 Medical oncology department, Centre François Baclesse, 14000 Caen, France

**Keywords:** Immunotherapy, Cancer, Cognition, Blood biomarkers, Preclinical model

## Abstract

**Background:**

The discovery of the importance of the immune system and its role in oncogenesis led to the development of immunotherapy, a treatment that represents a major advance in oncology management. Due to the recent nature of immunotherapy, little is known about its side effects and their impact on quality of life. To date, there is no published study that accurately assesses the impact of immunotherapy on cognition, mood and/or fatigue in patients treated for cancer, despite potential neurological toxicities. The purpose of this study is to prospectively assess the incidence of cognitive impairment and cognitive complaints among cancer patients naïve for immunotherapy without concomitant anti-cancer treatment.

**Methods:**

The Cog-Immuno trial is a multicentre longitudinal study addressing patients with cancer candidate to receive immunotherapy alone (*n* = 100). Immunotherapy treatment will include either anti-PD1/PDL1 or anti-CTLA4 monotherapy or combination therapy. Cognitive and quality of life assessment, electrocardiogram (ECG) and biological tests will be performed at baseline, thereafter 3, and 6 months after immunotherapy initiation. The primary endpoint is the proportion of patients treated by immunotherapy who will experience a decline in cognitive performances or in Montreal Cognitive Assessment (MoCA) score within 3 months after inclusion. Secondary endpoints concern: anxiety, depression, fatigue, clinical characteristics, biological data and neurophysiological measures (heart rate variability and hemispheric lateralization). A pre-clinical study will be conducted in cancer bearing mice receiving checkpoint inhibitors (ICI) with the evaluation of cognitive functions and emotional reactivity, collection of blood samples and investigation of neurobiological mechanisms from brain slices.

**Discussion:**

Assessing and understanding the incidence and the severity of cognitive impairment and its impact on quality of life in cancer patients treated by immunotherapy is a major issue. The results of this study will provide information on the impact of these treatments on cognitive functions in order to help the physicians in the choice of the treatment.

**Trial registration:**

NCT03599830, registered July 26, 2018.

**Protocol version:**

Version 5.1 dated from 2020/10/02.

## Background

Therapeutic advances now enable some patients to live for several years with cancer even in a metastatic situation and this is made possible by immunotherapy. However, cancer treatments are not without side effects and can be responsible, in some cases, for neurological toxicities. These toxicities are often expressed as cognitive impairment widely reported until now with chemotherapy. Thus, the impact of chemotherapy on cognition is now well documented but no study has yet explored the potential effect of anti-checkpoint inhibitors immunotherapy on cognitive functions.

Numerous studies have shown the impact of cancer treatments on cognition, especially chemotherapy. Patients treated, even for non-central nervous system cancers, mainly reported difficulties in remembering, thinking, concentrating or word finding [1]. These cognitive alterations are referred in the literature as Cancer-Related Cognitive Impairment (CRCI) [2].

Research has mainly focused on the impact of adjuvant chemotherapy on cognition in breast cancer patients. Longitudinal studies show that chemotherapy-induced cognitive decline occurs in 15-25% of patients [3]. Attention, executive functions, processing speed, episodic memory and working memory are mainly impaired. Furthermore, age seems to be a risk factor for cognitive decline after treatment and some chemotherapy molecules seem to have a greater deleterious impact on cognition [4].

Although the majority of studies have focused on the impact of adjuvant chemotherapy, there is also evidence of cognitive impairment in patients with metastatic disease, which may be greater than that seen in localized disease [5].

CRCI also seems to exist with the new targeted therapies which are becoming a major treatment in oncology. As an example, in patients treated for metastatic renal cancer, a prospective study shows a cognitive decline after initiation of antiangiogenic treatment in 31% of patients, without association with fatigue [6].

In addition to the other side effects of cancer treatment, cognitive difficulties have a negative impact on patients’ quality of life [7, 8] and can lead to a decrease in self-confidence in social settings or if a return to work is envisaged [7, 9, 10].

In the elderly, beyond their negative impact on quality of life, these cognitive disorders could also have an impact on the autonomy of patients [11]. It therefore warrants assessing and managing them in a geriatric population to avoid potential impact on autonomy.

### Immunotherapy in oncology

The discovery of the importance of the immune system and its role in oncogenesis led to the development of immunotherapy, a treatment that represents a major advance in oncology management.

In addition, with the breakthrough generated by immunotherapy based on CAR-T cells sometimes associated with strong toxicities, the passive specific immunization strategy is very promising. It involves monoclonal antibodies that inhibit immunosuppression induced by checkpoint inhibitors (ICI) such as cytotoxic T-lymphocyte antigen 4 (CTLA-4) or programmed cell death protein-1 (PD-1), antigens present on activated T-lymphocytes that negatively regulate their activation. PD-1 in particular is activated by its programmed cell death ligands 1/2 (PDL-1/L-2) expressed by tumor cells, allowing a local immunosuppressive action. The neutralization of CTLA-4 or PD-1, or even PD-L1, should promote an anti-tumor immune response [12]. Indeed, the use of antibodies that block the interaction of a ICI with its ligand induces complete and durable responses in patients with highly aggressive cancers such as melanoma, lung cancer and many others. The association with radiotherapy or chemotherapy also showed promising results but could potentiate neurological toxicities [13].

At the clinical level, the anti-CTLA-4 antibody (ipilimumab: Yervoy®) has been granted marketing authorisation for patients with metastatic melanoma. Anti-PD-1 antibodies (nivolumab: Opdivo®, pembrolizumab: Keytruda®) have demonstrated efficacy compared to standard therapy in metastatic melanoma, advanced lung cancer, metastatic renal cancer and bladder cancer [14–18]. In phase I and II trials, efficacy of these treatments has also been observed in other tumours: Hodgkin’s disease, head and neck cancers, gastric cancer, etc. [19].

Nevertheless, by modifying the immune balance, these treatments can be associated with the appearance of neurological toxicities (e.g. encephalopathy) and autoimmune side effects due to the lifting of the brake on the immune system induced by these molecules (mainly anti-CTLA-4 and anti-PD-1) [19]. These side effects are rare (1%) but probably underestimated and potentially serious [20]. The most frequent autoimmune side effect in the central nervous system is hypophysitis (18% of patients treated with anti-CTLA-4) with grade 3 or higher toxicity in 5% of cases. The most frequent manifestation is fatigue, which can lead to concentration problems. For anti-PD-1, specific autoimmune side effects are rare but severe when they occur [21]. These side effects usually occur within the first 2 months of treatment. However, due to the recent nature of immunotherapy, little is known about its side effects and their impact on quality of life [13, 22]. To date, only the findings from one pilot study were published on the impact of immunotherapy on cognition in patients treated for cancer [23]. In this small sample, cognitive decline, only assessed by cognitive screening tests, was more related to chemotherapy rather than immunotherapy. Thus, these first results should be completed in further studies with the use of several cognitive tests in a large sample, addressing a population naïve for immunotherapy without concomitant anti-cancer treatment.

### Neurophysiological factors and cognitive functions

Immunotherapy, now a standard part of cancer treatment, involves stimulating the patient’s immune system to improve its ability to recognise and attack cancer cells through inflammatory responses. Inflammatory reactions, while beneficial in the acute phase, can be harmful to the body if they become chronic [24]. The resulting pro-inflammatory environment leads to tissue damage [24], and when the brain is involved, the effects manifest themselves in the form of cognitive impairment [25–27]. Based on these findings, and on our previous studies on chemotherapy, plasma inflammatory biomarkers and cognition in animal models [28, 29], we hypothesise that the incidence, and even morethe extent, of cognitive impairment in patients treated with immunotherapy could be predicted from neuro-immunomodulatory factors.

How the central nervous system modulates the immune system depends on several factors, including hemispheric lateralization, which can be defined as the tendency for brain areas on one hemisphere to be more active than their counterparts on the opposite side. Differential activation of the right and left hemispheres has been shown to occur at several levels [30, 31], including the immune system [32–34]. More specifically, it has been shown that left hemispheric lateralization is generally associated with better immune performance [35]. Therefore, we believe that the possible use of hemispheric lateralization as a predictor of cognitive alterations deserves to be explored, as immunological effects resulting from immunotherapy, which may depend on hemispheric lateralization, may also have cognitive consequences.

Vagal tone is another factor of interest, as it reflects the activity of the vagus nerve, which plays a major role in the regulation of immunity and inflammatory processes [36–38]. Although various studies have cited vagal tone as a protective factor in various diseases [39], including cancer [40, 41], none to our knowledge have investigated the protective role it might play against cognitive impairment in immunotherapy. In order to examine this question, we propose to carry out electrocardiogram (ECG) in order to derive an indicator of vagal activity, namely heart rate variability (HRV) [42]. Indeed, HRV is related to multiple executive functions and to activity in brain regions responsible for executive functioning [43].

In addition to the above-mentioned neurophysiological factors, we are interested in examining whether it is possible to predict the impact of treatment on cognitive functions from the initial cognitive assessment of patients, and more specifically from their executive performance. As executive performance is underpinned by brain areas such as the dorsolateral prefrontal cortex [44–46] or the orbitofrontal cortex [47], both of which are involved in neuro-immunomodulation. Thus, it seems relevant to determine whether these measures can be considered not only as a means of monitoring immunotherapy-induced cognitive changes, but also as predictors of such changes.

Thus, two neurophysiological measures (HRV and hemispheric lateralization) will be performed in this study.

The question of the impact of these ICI on the neurobiological mechanisms remains to be established and only a preclinical model will be able to address this. A recent study showed that anti-CTLA-4 immunotherapy combined with peripheral targeted radiotherapy resulted in impaired anxiety and cognitive functions associated with neuro-inflammation and microglial activation in mice [48], but the mechanisms linking tumor and immunotherapy are not understood. Thus, we connect to the current protocol a preclinical behavioral mouse model to identify the existence of inflammatory biomarkers associated with cold or hot non-brain cancers, which could, in combination with ICI immunotherapy help to the recruitment of immune cells to the brain and/or stimulate neural pathways and promote neuro-inflammation or local lesions, associated with disturbances in emotional and/or cognitive functions. Thus, while several potential biomarkers identified as predictors of immunotherapy-associated adverse events have been identified (CYTOX score) but not yet prospectively validated, we will prepare a biobank of peripheral blood mononuclear cell (PBMC) in the cohort of immunotherapy patients, but also in mice bearing cancers and treated with ICI.

Together, we aim to evidence the impact of ICIs on cognitive functions and to characterize a signature of biomarkers predictive of the occurrence of neurological toxicities.

## Methods/design

**Fig. 1:**
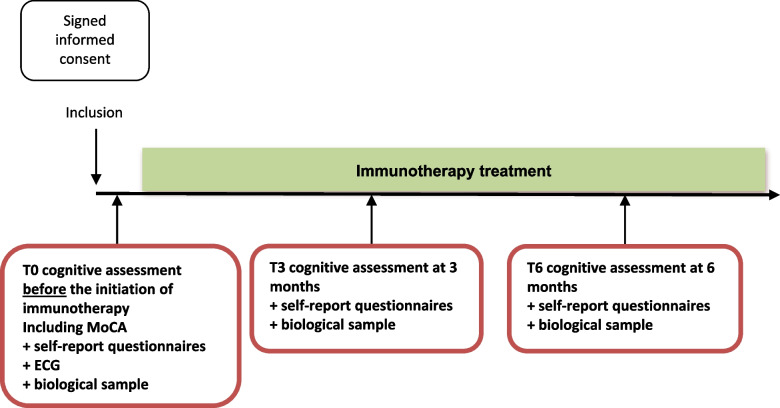
Study flowchart of the Cog-Immuno study

The Cog-Immuno study is an on-going multicentre longitudinal study (Fig. 1). The Cog-Immuno protocol and this manuscript have been written in accordance with standard protocol items, namely recommendations for interventional trials (SPIRIT).

### Primary outcome

The primary objective of this study is to prospectively assess the incidence of cognitive impairment and cognitive complaints among cancer patients naïve for immunotherapy without concomitant anti-cancer treatment. The primary endpoint is the proportion of patients treated by immunotherapy who will experience a decline in cognitive performances (at least for one cognitive domain) or in MoCA score within 3 months after inclusion. It will be presented with its exact confidence interval at the 95% confidence level.

### Secondary outcomes

The secondary objectives are to assess:

- The relationship between objective cognitive impairment and anxiety, depression, and fatigue,
- The relationship between objective cognitive impairment and cognitive complaints,
- The relationship between cognitive functioning (objective and subjective) and clinical characteristics such as cancer stage, comorbidities, comedications…
- The relationship between cognitive impairment and biological data,
- The incidence or the severity of cognitive impairment induced by immunotherapy based on neurophysiological measures and cognitive ones.

### Study population

The Cog-Immuno trial addresses cancer patients naïve for immunotherapy without concomitant anti-cancer treatment.

Inclusion criteria are: 18-year old or more, patient with cancer who is to be started on immunotherapy alone, immunotherapy treatment will include either anti-PD1/L1 or anti-CTLA4 monotherapy or combination therapy, patient may have received other anti-tumor treatments other than immunotherapy but these must be discontinued at the time of initiation of immunotherapy, performance Status ≤2, patient with a minimum of education level “end of primary education”, patient affiliated to a social security regimen, patient having signed the written informed consent to participate in the study.

Non-inclusion criteria are: previous treatment with immunotherapy, other ongoing anti-tumor treatment, primary cancer of the central nervous system or symptomatic and uncontrolled brain metastasis(es), alcohol abuse or drug use, poor French language fluency, severe visual and/or auditory deficits, patient deprived of liberty or under guardianship, patient unable to undergo the study follow-up for geographical, social or psychopathological reasons.

### Study sites

The list of study sites is indicated on https://clinicaltrials.gov/ct2/show/NCT03599830. The participation of 4 French centres is planned tp achieve the required sample size: the Cancer Comprehensive Centre François Baclesse, and the University Hospital Centres from Amiens, Caen and Lille.

### Study experimental plan

The study will be proposed to the patients who meet the eligibility criteria. An explanation of the study and an information note will be given to them. Patients will be enrolled in the study once provided their written informed consent.

The patients will be recruited in the participating centres over 48 months. Their participation will last 6 months.

### Study assessments

The study flow-chart and overview of study assessments (cognitive tests, self-report questionnaires for quality of life evaluation, and biological tests) are indicated in Fig. 1 and Table 1. Assessments will be conducted, once signed the consent form, at inclusion - baseline (T0) - (before the start of immunotherapy or within 7 days after the start of immunotherapy), 3 months (±15 days; T3), and 6 (±15 days; T6) months after the immunotherapy initiation.

**Table 1:** Overview of the Cog-Immuno study assessments

Assessment	T0^c^	T3 3 months	T6 6 months
**Signed informed consent**	✓		
**Cognitive assessment**^**a**^			
-MoCA^2^	✓	✓	✓
If normal MoCA score^b^:			
-Hopkins verbal learning test	✓	✓	✓
-Digit span (WAIS-IV)	✓	✓	✓
-Trail Making test	✓	✓	✓
-Stroop	✓	✓	✓
-Symbol search (WAIS-IV)	✓	✓	✓
-Verbal fluencies	✓	✓	✓
-Cancellation (WAIS-IV)	✓	✓	✓
-Line bissection	✓		
**Self-report questionnaires**			
FACT-Cog, FACIT-F, HADS	✓	✓	✓
**ECG**^**d**^	✓		
**Biological sample**			
Biological tests^e^	✓	✓	✓
Research specific blood samples^f^	✓	✓	✓

^a^ Cognitive assessments by a neuropsychologist

^b^ At baseline, the MoCA score should be above threshold value based on age and education level normative data (Table 2) to realize the complete cognitive battery. In the case of MoCA score below threshold value, only MoCA and self-report questionnaires will be proposed to the patient for baseline, 3 and 6 months assessments

^c^ Before the start of the treatment or within 7 days after the start of immunotherapy

^d^ Standard ECG required for all participating centres. A 5-minute ECG will be performed for centres with a specific device. A centralized review of the ECGs is planned

^e^ CBC-platelets, sodium, potassium, ALKP, ASAT, ALAT, GGT, total bilirubin, creatinin, albumin, CRP and TSH

^f^ For patients with specific informed written consent for constitution of a biobank of PBMC, serum and plasma from blood samples: 2 CPTTM (2X4 mm) and 1 BD vacutainer serum tube (5 ml)

At inclusion, previous medical history will be reported as well as relevant medications (psychotropic, opioids...). The cognitive evaluation will first be based on the realization of the Montreal Cognitive Assessment (MoCA): the score should be above threshold value based on age and education level normative data [49] (Table 2) to perform the complete battery of cognitive tests. In the case of a MoCA score below threshold value, indicating overall cognitive impairment, the cognitive evaluation will be restricted to the MoCA and self-report questionnaire at baseline, 3 and 6 months assessment.

**Table 2:** Threshold value of the MoCA according to GRECOVASC normative data [49]

Age	40-60 years			61-70 years			71-85 years		
Education level^a^	1	2	3	1	2	3	1	2	3
Threshold value to perform all cognitive tests of the study (5th centile)	21	23	24	21	22	23	20	22	23

^a^Level 1: low

Level 2: medium

Level 3: high

#### Objective cognitive assessment

Objective cognitive functions will be assessed by the International Cognition and Cancer Taskforce (ICCTF) recommended battery of tests [50]. The full evaluation will take less than 1 hour and will be performed by a neuropsychologist.

Global cognitive efficiency will be assessed by the MoCA, a rapid screening instrument for cognitive impairment [51].

The main explored cognitive domains are the most impaired by cancer treatments [2]: executive functions (Trail Making test, Stroop, verbal fluencies [52]), attention (cancellation, Weschler Adult Intelligence Scale (WAIS)-IV [53]), information processing speed (Symbol search WAIS-IV [53]) and memory (Hopkins verbal learning test [54], digit span [53]). Line bisection [55] will be used to assess hemispheric lateralization [56].

As above mentioned, this full battery of cognitive tests will be performing only by patients obtained normal range MoCA score.

#### Quality of life assessment

We will use validated self-report questionnaires to evaluate cognitive complaints (Functional Assessment of Cancer Therapy Cognitive Scale: FACT-Cog [57]), depression and anxiety (Hospital Anxiety and Depression Scale: HADS [58]) and fatigue (Functional Assessment of Chronic Illness Therapy-Fatigue: FACIT-F [59]).

#### Neurophysiological measures

Two neurophysiological measures (HRV and hemispheric lateralization) will be performed in this study.

Hemispheric lateralization will be estimated with the line bisection test [55].

Heart rate variability (HRV) will be estimated from an electrocardiogram (ECG). A standard ECG device is required for all participating centres. A 5-minute ECG will be performed for centres with a specific digital ECG device. A centralized review of the baseline ECGs is planned, using ECG paper outlines from centres without digital ECG device, and digital ECG copies. The resulting data will be converted into time domain (SDNN, RMSSD) and frequency domain (HF, LF, LF/HF) measurements using MatLab software and a set of adapted algorithms [60–62].

#### Biological tests and biological collection

Standard of care will be done by assessing from blood: CBC-platelets, sodium, potassium, ALKP, ASAT, ALAT, GGT, total bilirubin, creatinin, albumin, CRP and TSH.

Optional specific blood samples (about 15 mL) for the further research will be collected at T0, T3, T6 for constitution of a biobank of PBMC, serum and plasma from blood samples of patients who have provided additional specific informed consent. Thus, at each collection time, 2 CPTTM (2X4 mm) and 1 BD vacutainer serum tube (5 mL). The CPT samples will be immediately sent to the Inserm U1245 laboratory (Rouen, France) for PBMC and plasma preparation under sterile condition while serum will be collected after centrifugation (3000 rpm, 10 min). PBMC will be stored in liquid nitrogen while plasma and serum will be kept frozen at − 80 °C.

These anonymized samples will be used for further hormonal, cytokine and genetic analyses. The samples will be stored in a secure area (CBG laboratory, Inserm research Unit, Rouen) with restricted access in accordance with the regulations in force.

### Statistical design overview

#### Sample size determination

To our knowledge, there is no published study about the estimation of the incidence of cognitive impairment in cancer patients treated with immunotherapy. We propose to carry out the present study, assuming that about 50% of the patients will develop cognitive impairment under immunotherapy. Using a 95% confidence interval with a width of 0.2 for small sample, it is necessary to include 93 assessable patients. To take into account lost to follow-up, we plan to enrol 7 additional patients, for a total of 100 patients.

#### Statistical analyses

Exploratory analyses of the data will provide, for quantitative variables, the mean, standard deviation, median, quartiles, and number of missing values; for qualitative variables, we will calculate the frequencies and their 95% confidence intervals. The demographic and clinical characteristics of the patients will be described. The estimation of the incidence of the cognitive impairment will be described according to the characteristics of the patients. When possible, statistical tests (non-parametric) will be used to estimate the association between different factors and the occurrence of cognitive impairment. A two-sided alpha risk of < 0.05 will be considered significant. We will measure the association between cardiac variability, hemispheric lateralization and psychological variables on the one hand, and cognitive alterations on the other, using a hierarchical regression analysis, after taking into account the initial cognitive assessment and prognostic factors (age, cancer stage, etc.).

### Ancillary pre-clinical study

A complementary pre-clinical study will be conducted using mouse behavioural preclinical models. C57B/l6 mice bearing different immunogenic murine cancers (melanoma B16F10 and B16F10-ova cells; murine colon carcinoma MC38 cells) will be treated by three injections (1/week) of murine control IgGs, anti-PD-1 or anti-PDL-1 from day 3 after cancer cells inoculation. Activity, cognitive performances and emotional reactivity using a battery of behavioral tests will be assessed during the course of the treatment. Given the neuropsychological characteristics of cancer patients and the neurobiological impairment sometimes observed in treated patients, our behavioural analysis will mainly focus on tests that highlight an alteration of the hippocampus and the pre-frontal cortex. For emotional reactivity, tail suspension (TST, FST, elevated cross maze; cognition, Morris pool, object recognition), behavioural tests will be performed over 5 weeks overall.

In order to investigate whether certain alterations in cognitive functions or emotional responses are associated with neuro-inflammation and/or changes in the cerebral vascular network, the levels of intraparenchymal cytokines TNF-α, IL-1β, IL-17, IL-6 and INF-γ will be analysed by Elisa and quantitative PCR (one cerebral hemisphere will be sampled for protein lysate preparation, and the other hemisphere for mRNA preparation). From fresh brain samples including choroid plexuses, leukocytes will be isolated by flow cytometry using antibodies myeloid cells (resident microglia and macrophages) and against T lymphocytes (CD4 and/or CD8), NK (DC56) and B cells (CD19), as well as granulocytes and monocytes. By immunohistochemistry, microglial reactivity and reactive astrogliosis will also be investigated in particular in the vascular network, in the hippocampus and/or the pre-frontal cortex. Neuronal degeneration using Fluorojade-c + labelling in the brain area innervated at least in part by the vagus nerve, in mice bearing the most immunogenic B16F10-Ova and MC38 cancers and treated by ICI, will be tested. At the vascular level, by double labelling, endothelial reactivity will be checked using antibodies directed against adhesion proteins ICAM, VCAM, selectins and certain integrins, and the sites of leukocyte infiltration will also be sought at the edges of the cerebral vasculature.

Then, from plasma samples and brain extracts, we will search for plasma biomarkers (cytokine assays) and neurobiological mechanisms such as cerebral vascularisation, neuro-inflammation (Western blot, flow cytometry, immunohistochemistry) and electrical activities (patch-clamp on slice) associated with potential disturbances of emotion and/or cognitive functions in animals treated with immunotherapy targeting PD-1 or PD-L1.

### Data management

A Web Based Data Capture (WBDC) system will be used for data collection and query handling. The investigator will ensure that data are recorded on the eCRFs as specified in the study protocol and in accordance with the instructions provided.

The investigator ensures the accuracy, completeness, and timeliness of the data recorded and of the provision of answers to data queries according to the Clinical Study Agreement. The investigator will sign the completed eCRFs. A copy of the completed eCRFs will be archived at the study site.

### Withdrawal from study

The reasons for why a patient may discontinue to participate to the study include the following circumstances:

- Immunotherapy treatment discontinuation, whatever the reason (unacceptable toxicity, disease progression…),
- Patient’s decision (the data already collected during the search can be kept and exploited unless the patient opposes it),
- Intercurrent illness or other reason that requires stopping participation to the study
- Patient lost to view,
- Investigator’s decision.

## Discussion

Due to the recent nature of immunotherapy, little is known about its side effects and their impact on quality of life and, to date, there is no published study that accurately assessed the impact of immunotherapy on cognition in patients treated for cancer despite potential neurological toxicities.

The ancillary pre-clinical study will also help to understand the physiopathological mechanisms of cognitive impairment based on plasma biomarkers, neuro-inflammation and/or changes in the cerebral vascular network.

The objectives the Cog-Immuno study are in line with French national priorities for cancer research, in particular Axis 2 of the new Ten-Year Cancer Strategy, which aims to “limit the cancer treatment side-effects and improve quality of life” by “improving the post-cancer period”.

## Conclusion

Evaluating and understanding the incidence and the severity of cognitive impairment in patients treated by immunotherapy is a major issue. The Cog-Immuno study results will provide information for patients on impact of immunotherapy on cognitive functions in order to help the physicians in the choice of the treatment and could promote the development of cognitively safe treatments.

## Data Availability

This study is ongoing.

## Abbreviations

ECG: Electrocardiogram
CAR: Chimeric antigen receptor
eCRF: Electronic case report form
ICIs: Immune checkpoint inhibitors
CRCI: Cancer-Related Cognitive Impairment
FACT-Cog: Functional Assessment of Cancer Therapy Cognitive Scale
FACIT-F: Functional Assessment of Chronic Illness Therapy-Fatigue
HADS: Hospital Anxiety and Depression Scale
HRV: Heart rate variability
ICCTF: International Cognition and Cancer Taskforce
MoCA: Montreal Cognitive Assessment
SPIRIT: Standard Protocol Items, Recommendations for Interventional Trials
WAIS: Weschler Adult Intelligence Scale
